# One-Piece Mini Dental Implant-Retained Mandibular Overdentures: 10-Year Clinical and Radiological Outcomes of a Non-Comparative Longitudinal Observational Study

**DOI:** 10.3390/jfb15040099

**Published:** 2024-04-11

**Authors:** Nicole Schenk, Hristina Bukvic, Martin Schimmel, Samir Abou-Ayash, Norbert Enkling

**Affiliations:** 1 Department of Reconstructive Dentistry and Gerodontology, School of Dental Medicine, University of Bern, 3010 Bern, Switzerland; nicole.schenk@unibe.ch (N.S.); hristina.bukvic@unibe.ch (H.B.); martin.schimmel@unibe.ch (M.S.); norbert.enkling@unibe.ch (N.E.); 2 Division of Gerodontology and Removable Prosthodontics, University of Geneva, 1205 Geneva, Switzerland; 3 Department of Prosthodontics, Preclinical Education and Dental Materials Science, Medical Faculty, University of Bonn, 53113 Bonn, Germany

**Keywords:** bone level alterations, bone level changes, implant success, implant survival, mini dental implants, narrow-diameter implants, elderly patients, overdenture

## Abstract

This study presents the first 10-year follow-up investigation of the implant survival and peri-implant outcomes of one-piece mini dental implants (MDIs) retaining mandibular implant overdentures (IODs), including marginal bone level alterations (ΔMBLs), clinical peri-implant parameters, and complications. Twenty participants with horizontally atrophied mandibles received complete dentures and four MDIs (diameter 1.8 mm) at baseline. The dentures were converted into IODs with O-ring attachments. The 10-year follow-up comprised a radiological assessment of ΔMBLs, peri-implant parameters, as well as biological and technical complications. Results from a 10-year follow-up of 14 participants showed a 100% implant survival rate for all 56 implants. The mean ΔMBL after 10 years was −1.12 ± 0.80 mm, with 49 implants classified as successful (ΔMBL < 2 mm) and 7 implants with satisfactory survival (ΔMBL 2–4 mm). Time after implant placement significantly influenced ΔMBL, with stable MBLs after 5 years. The prosthetic survival rate after 10 years was 93%. ΔMBLs were not influenced by implant position or gender but were significantly smaller in subjects older than 65 years. Conclusively, one-piece MDIs with O-ring attachments offer a reliable treatment option for horizontally atrophied mandibles after 10 years, with high implant and prosthetic survival rates, potentially benefiting from advanced age regarding peri-implant bone stability.

## 1. Introduction

Complete edentulism affects all aspects of oro-facial function [1]. The reduced ability to effectively chew, speak, and swallow [2,3] results in a reduced oral health-related quality of life (OHRQoL) [4].

Rehabilitation of the edentulous mandible with a removable complete denture (CD) is often considered difficult, especially after severe bone atrophy, and may result in an unfavorable treatment outcome [5]. The use of dental implants has proven to be an effective method to significantly improve oral function and OHRQoL [6,7]. In this context, two-implant-retained mandibular overdentures (IODs) have been introduced as the standard implant treatment option for the rehabilitation of an edentulous mandible [8,9]. However, the bone resorption that occurs after tooth loss often results in insufficient bone volume, which often makes the placement of standard implants (>3.5 mm) difficult or even impossible. Especially in medically compromised patients of advanced age, the bone augmentations required prior to implant placement lead to a higher risk of biological complications [10,11]. Therefore, different treatment approaches have been proposed to avoid extensive bone augmentation. In addition to the placement of shorter implants [12], which have only been described in posterior jaw areas [13], implants with a diameter of ≤3.5 mm (narrow diameter implants; NDIs) have been increasingly used in recent years [14,15]. In horizontally atrophied edentulous mandibles with a knife-edge ridge form, in which treatment with standard diameter implants is not possible due to insufficient alveolar ridge width, one-piece mini dental implants (MDIs; diameter < 2.5 mm) showed significant advantages [15]. The majority of commercially accessible MDIs are designed for the retention of overdentures, with the retentive element integrated into the implant body. Originally developed for the temporary stabilization of overdentures, MDIs had demonstrated unexpected osseointegration properties, thereby establishing their viability for long-term applications. Titanium (Ti) plays an important role here, as it is known for its good corrosion resistance and high stability because of the stable and inert oxide layer and is widely regarded as the most biocompatible metal [16]. As commercially available pure titanium has limited mechanical strength, alloying or advanced surface treatment techniques are used to improve the mechanical properties of Ti [16,17]. Since MDIs have an increased risk of fracture due to the reduced diameter, titanium alloys (Ti-6Al-4V, titanium–zirconium alloys) are preferred for the production of MDIs. However, despite the above-mentioned advantages, a recent meta-analysis showed a significantly lower survival rate of MDIs (94.7 ± 5%) compared with standard diameter implants [14].

Among other attachments, ball/O-rings are commonly used as retentive elements for MDIs. Because of the lack of occlusal stops, the O-rings act as load-damping elements and prevent the direct transmission of masticatory forces into the surrounding bone, and the forces are still transferred to the surrounding mucosa, similar to the load distribution in CDs [18]. While only short- and medium-term results are available in addition to results from retrospective studies [19,20,21,22], there is no available evidence of the long-term survival of MDIs. 

Therefore, the present longitudinal observational clinical study analyzed the 10-year implant survival/success rates, marginal bone level alterations (ΔMBLs), and peri-implant outcomes of patients treated with mandibular overdentures retained by four titanium MDIs. The null hypothesis (H0) was that there would be no difference in ΔMBLs among the first 5 years of follow-up compared with ΔMBLs between 5 and 10 years. Furthermore, the potential effects of patient gender, implant position, time after implant placement, and age on ΔMBLs, as well as probing depths (PDs) and bleeding on probing (BOP), the presence of plaque and keratinized mucosa (KM), and the number of technical and biological complications of MDIs and IODs, were examined.

## 2. Materials and Methods

The present study was a non-comparative longitudinal observational study with a follow-up period of 10 years. In addition to the approval of the study protocol by the Cantonal Ethics Committee of the Canton of Bern (CEC No. 26/10, approve date: 5 July 2010), this study complies with the ethical standards of the Declaration of Helsinki as well as the national legal and regulatory requirements [23]. All participants voluntarily participated in this study and gave written informed consent before the start of this study.

### 2.1. Patient Enrolment

Details of the inclusion and exclusion criteria were described in more detail previously in the 1-year follow-up report [24]. In summary, all participants recruited at the School of Dental Medicine, University of Bern (Switzerland), between November 2010 and March 2012 were selected according to the following inclusion criteria:

- Complete edentulism (minimum duration of 6 months);
- Interforaminal bone dimension of at least 4 mm clinical width (clinical examination with a periodontal probe) and a minimum height of approximately 13 mm (assessed by two-dimensional X-ray examination);
- Bone augmentation required for standard diameter implant placement;
- Good general health (ASA classification 1 or 2);
- Not dependent on care.

The main objective was to improve the retention of mandibular prostheses using implants.

The exclusion criteria were the presence of medical conditions that constitute a contraindication to implantation/therapy and the use of medications that may affect bone metabolism. Furthermore, patients with medication abuse and dental anxiety were excluded.

The study group included fifteen females and five males (total *n* = 20) with a median age of 65.5 years (range 41–87 years) at the time of implant placement. 

### 2.2. Study Procedures

**Figure 1:**
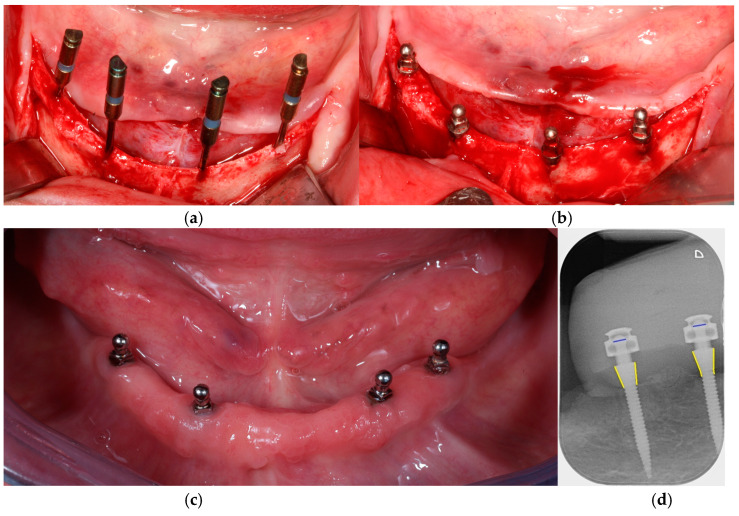
Overview of a patient case: (**a**,**b**) occlusal view of the mandible with four mini dental implants (MDIs) during surgery, demonstrating the horizontally atrophied mandible with a knife-edge ridge; (**c**) MDIs at the 10-year follow-up examination; and (**d**) radiological evaluation of marginal bone levels (MBLs) at regions 42 and 44 at the 10-year follow-up. The blue lines represent the 1.8 mm diameter balls that were used to calibrate the subsequent measurements; the yellow lines represent the measured distances (MBLs) along the implant contours.

All participants received new CDs at baseline (BL), which they had worn for at least 12 weeks prior to the surgical intervention. The posterior teeth (Condyloform^®^ II NFC+, Candulor Dental GmbH, Glattpark, Switzerland) were set up according to the Gerber concept with a bilaterally balanced, lingualized occlusion [25] and ended with the first molar. With the aim of stabilizing the existing CDs, four one-piece titanium MDIs (titanium grade 23 (TiAl6V4 ELI), MDI^®^ system 3M ESPE, distributed since 2016 by Condent GmbH, Hannover, Germany) were placed freehand after raising a full-thickness flap. Participants were prescribed prophylaxis one hour before the surgical procedure (2 g Augmentin or 600 mg Clindamycin). In all patients, the interforaminal region showed predominantly cortical, tapering severely atrophic bone (bone type D1) [26]. The implants were placed as parallel as possible in the interforaminal area. The junction between the rough and machined implant surfaces was placed about 1 mm subcrestally. All implants had a diameter of 1.8 mm and a length of 13 or 15 mm. The posterior implants were placed 5 mm anterior to the mental foramen, and it was aimed to achieve a uniform distance of approximately 15 mm between all implants (Figure 1a–c).

**Figure 2:**
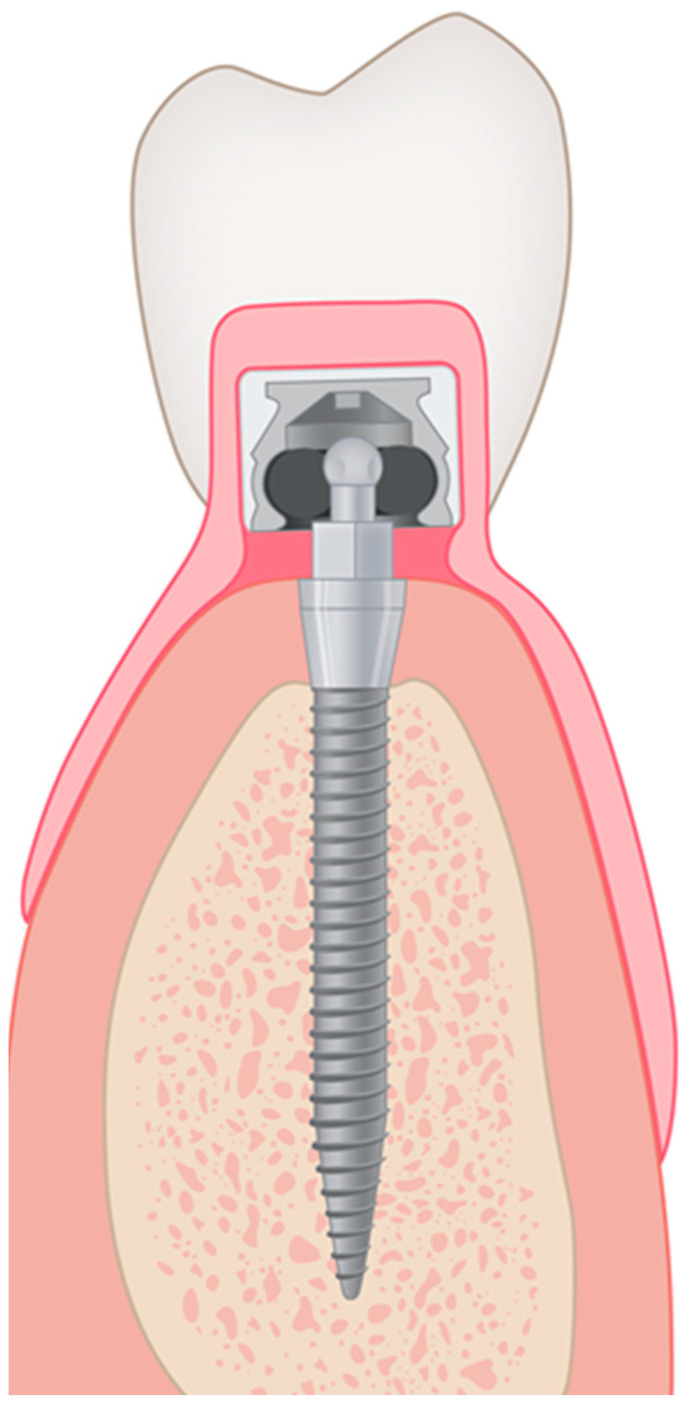
Schematic drawing of an MDI implant with the corresponding attachment system.

Immediate loading of the implants was performed within 24 h after implant placement using the system’s matrices. The MDI^®^ system (Figure 2) comes with different metal housings regarding retention and size as follows: standard (MH-1), micro (MH-2), or o-cap (MH-3) matrices including nitrile rubber O-rings with a diameter of 3.5 mm. Polyether reline impressions (Impregum, 3M ESPE, Seefeld, Germany) were taken, and subsequently, titanium MH-2 matrices (MDI, 3M ESPE, Seefeld, Germany) with a housing diameter of 4.3 mm and a height of 3.3 mm were integrated into the existing CD in an indirect procedure. Patients received the final prostheses on the same day by the prosthodontist, who also recorded the BL. All further examinations were performed by an independent prosthodontist who was not involved in the previous treatment procedures.

### 2.3. Outcome Measures

#### 2.3.1. Radiographic Analysis of Peri-Implant Bone Levels

**Figure 3:**
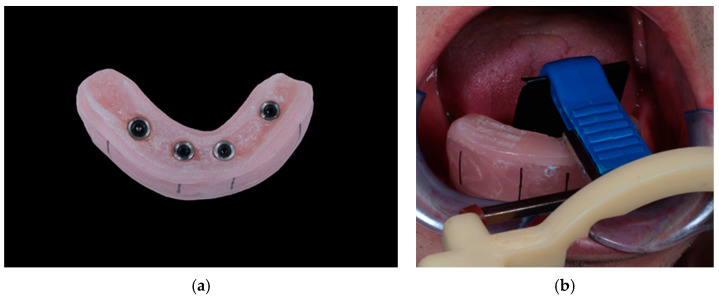
View of a radiological splint: (**a**) basal view of a radiological splint, including MH2 matrices and O-rings, and (**b**) clinical set-up for standardized radiograph recording.

Starting from BL (time point of immediate loading), radiological and clinical examinations were performed after 3 months, 6 months, 12 months, 3 years, 5 years, and 10 years. Despite the evaluations at BL, all further evaluations were performed by an independent prosthodontist who was not involved in the surgical or prosthetic study procedures. At all appointments, including the BL visit, digital radiographs were recorded using the paralleling technique with custom-made acrylic splints (Figure 3a,b). These were created, based on the relining impressions, to ensure the consistent positioning of X-ray film holders for each participant.

The radiographic images were analyzed by two calibrated clinicians independently at two different time points, using a software program (ImageJ 2, National Institutes of Health; Software version 1.53) at 10-fold magnification. For calibration purposes, the two clinicians analyzed 20 randomly selected X-rays together. To obtain correct dimensions for subsequent measurements, calibration was performed before each measurement using the male part of the implants (i.e., the ball) with a diameter of 1.8 mm. To be consistent with the previous study, the distance from the first bone-to-implant contact to the implant shoulder was measured along the outer implant contour at both the mesial and distal aspects (Figure 1d). 

For each implant at each time point, the MBL (the average distance from the implant shoulder to the first bone-to-implant contact) was determined by averaging eight measurements (two from each clinician at both the mesial and distal aspects). ΔMBLs were then calculated by subtracting the follow-up visit MBL values from the baseline values.

#### 2.3.2. Clinical Parameters

The following clinical parameters were used for the evaluation of peri-implant conditions:

- mPI: modified Plaque Index [27];
- PPD: peri-implant probing pocket depth: measured to the nearest millimeter with a Hu-Friedy PGF–GFS periodontal probe (Hu-Friedy, Chicago, IL, USA);
- The occurrence of subsequent bleeding (yes/no);
- The presence of KM at the lingual and buccal aspects.

In addition, the prostheses were examined, and the patient records were searched for the following technical complications:

- Matrix loosening;
- Denture cracking and fractures;
- The need for relining;
- Any signs of wear at the male part.

#### 2.3.3. Definition of Implant Survival, Success, and Denture Survival

Implant survival was defined as the implant being present at the time of examination. Implant success was defined by the criteria established at the International Congress of Oral Implantologists (ICOI), considering the presence of ΔMBLs, mobility, pain, exudate, and probing depth [28]. If the radiographic bone loss was less than 2 mm and no pain, no exudates history, and no implant mobility was observed, the implants were considered successful. Implants that showed radiographic bone loss between 2 and 4 mm, but the other criteria were unchanged, were considered as satisfactory survival. The survival rate of the overdenture was defined by the presence without the need for remaking.

### 2.4. Statistical Analyses

Analyzing ΔMBLs and the peri-implant parameters descriptively, mean values and standard deviations (SDs) were calculated. All analyses were performed for each implant site at every time point separately. Furthermore, for some subgroups, median values and interquartile ranges (IQRs) were given. In addition, boxplots were utilized to illustrate the distribution of ΔMBLs in both age groups at each time point.

A random-effects linear regression analysis was performed to estimate the potential impact of implant position, patient age, patient gender, and duration following implant placement on ΔMBLs. The Wald Test was used to test the null hypothesis of no impact. Furthermore, to evaluate the interrater agreement and intra-rater reliability for the performed MBL measurements, Bland–Altman statistics were used. ΔMBL was measured per implant, and all statistical analyses were on the implant level. 

The statistical analyses were performed using Stata/IC software (Version 14.2 for Windows) with the alpha level set to 0.05.

## 3. Results

### 3.1. Description of Participants

A total of 20 participants including five men and fifteen women participated in this study. The median age at the time of implant placement was 65.5 years (min = 41 years, max = 87 years).

While all participants attended their 1-year recall visit, one of the participants could not be clinically examined at the end of the 5-year period because of health problems. 

At the end of the 10-year study period, five more patients dropped out because of inaccessibility via phone or mail. Within the remaining 14 participants, 5 of them were older (older group; median age: 82 [IQR:11] years; four females, one male) and 9 of them were younger than or equal to 65 years old at BL (younger group; median age: 74 [IQR:11] years; six females, three males). The mean follow-up time for the participants at the 10-year follow-up appointment was 10.3 years (min = 9.2 years, max = 11 years).

### 3.2. Marginal Bone Level Alterations (ΔMBLs)

**Figure 4:**
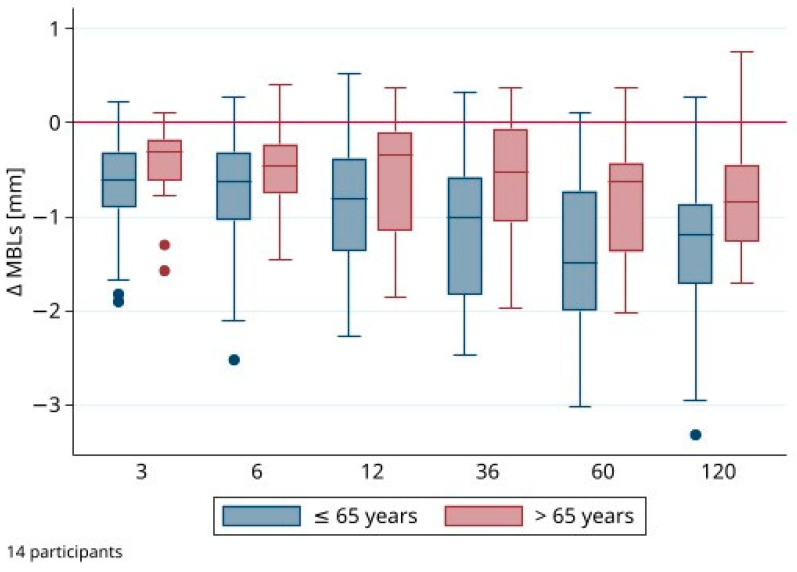
Distribution of marginal bone level alterations (ΔMBL) of all implants (*n* = 56) per age group.

A total of 56 implants were evaluated at each time point. The mean ΔMBLs were −1.42 ± 0.88 mm at position 34, −1.06 ± 0.53 mm at position 32, −1.04 ± 0.58 mm at position 42, and −0.95 ± 1.08 mm at position 44 after 10 years. Table 1 provides an overview of the mean ΔMBLs for each implant site and each time point during follow-up. Analyzing the ΔMBLs of the implant sites at 6, 12, 36, 60, and 120 months showed significant differences between the time points and baseline (*p* < 0.001, global test). There was no significant influence of the implant position (*p* = 0.724) or patient gender (*p* = 0.309) on ΔMBLs. Yet, ΔMBLs were significantly higher in participants from the younger group compared with those from the older group (*p* = 0.009). A second regression analysis was performed, taking only the ΔMBL after 10 years into consideration. With restriction to the last time point, there was also a significant difference between the two age groups in terms of ΔMBL (*p* = 0.031; Figure 4). Comparing the ΔMBLs from baseline to 5 years to those from 5 to 10 years, the MBLs remained stable after 5 years (mean alterations after 5 years: 0.06 ± 0.64 mm). While no influence of implant position (*p* = 0.299) and patient gender (*p* = 0.593) was found, ΔMBLs were significantly smaller in subjects older than 65 years (*p* = 0.004).

The individual measurements of the two clinicians showed an estimated mean interrater difference of 0.03 mm (95% CI: [0.01; 0.06]; *p* = 0.013) and an intra-rater difference of 0.01 mm (95% CI: [−0.01; 0.02]; *p* = 0.456).

### 3.3. Clinical Evaluation

The implant survival was 100%. No implants presented signs of pain, mobility, or exudate. The implant success rate showed an 87.5% successful (ΔMBL < 2 mm) and a 12.5% (ΔMBL of 2–4 mm) satisfactory success rate according to the Health Scale for dental implants [26]. The number of implants was then categorized according to these success criteria and the presence of KM (Table 2), indicating no influence of KM on the success ratings.

Table 3 provides an overview of the evaluated peri-implant parameters during the 10-year follow-up period. Compared with the 5-year follow-up, all parameters were increased at the 10-year follow-up. Looking at the BOP-positive sites in the present study, in all except for one patient, at least one implant site was BOP-positive (Table 3).

The overall survival for MDI-retained mandibular overdentures after 10 years was 93%. During the 10-year follow-up appointment, one set of dentures had to be replaced because of functional problems, specifically occlusal wear and inadequate base fitting. Regarding technical complications during the second study period, such as denture fractures, matrix loosening, or replacement of the O-ring inserts, no reliable statement can be made because the regular recall visits of the patients were performed in private practice. However, during the 10-year follow-up appointment, no severe prosthetic treatment need was observed. All O-rings were replaced as part of the study protocol during the follow-up assessments. No wear of the ball attachments for any of the implants could be detected by visual inspection, none of the metal housings had to be replaced, no fractures occurred, and only one prosthesis had to be relined.

## 4. Discussion

The present study assessed MDI and prosthetic survival and success, the temporal sequence of ΔMBLs, and clinical peri-implant parameters after 10 years of function. Within the current study protocol, the MDIs had a 100% survival rate, whereas the prostheses demonstrated a 93% survival rate. The implants were rated 87.5% successful with a 12.5% satisfactory success rate, which was related to both radiographic peri-implant bone loss and soft-tissue parameters (BOP, PD). The null hypothesis (H0) that there would be no difference in ΔMBLs among the first 5 years of follow-up compared with ΔMBLs between 5 and 10 years was rejected. The MBLs remained constant in the second evaluation period. The ΔMBLs were not influenced by the implant location, patient gender, or the presence of keratinized mucosa. Both overall and after the 10-year follow-up, ΔMBLs were found to be significantly smaller in the older compared with the younger group.

The demonstrated 100% survival rate is consistent with previously published systematic reviews that reported survival rates of 94.2–100% [29] and 98% [30]. The use of MDIs to retain IODs was also investigated in another systematic review [31], which reported a survival rate of 92.32% after a mean follow-up period of 30 months. Although the survival rates of MDIs were described to be comparable to those of standard implants [30,32], another systematic review indicated significant differences between the three categories of narrow diameter implants (NDIs) compared with standard diameter implants [14]: While NDIs of Categories 2 and 3 had comparable survival rates, NDIs of Category 1, with a diameter of <2.5 mm or less, showed a noticeably increased risk of implant loss, with an odds ratio of 4.54 (CI: 1.51–13.65). Although novel mini implant systems show promising short-term data [33], it should be noted that none of the above-mentioned systematic reviews included long-term data. The 100% implant survival rate after 10 years in the present study could be related to the O-rings that reduce the force on the implants. Unfortunately, the prosthetic connection between the implants and the overdentures was not part of any of the previously mentioned reviews.

Considering the currently accepted peri-implant parameters [28,34,35] as indicators for implant success, the present study showed an implant success of 87.5% with a mean ΔMBL of −1.12 ± 0.80 mm. Although patients with unfavorable anatomical conditions and poor bone quality were included in the present study, their average ΔMBLs after 5 years [22] were within clinically acceptable limits (−1.18 mm ± 0.79). Regarding the 5- to 10-year follow-up, a stagnation of peri-implant bone loss was observed (0.06 ± 0.64). According to the criteria of Albrektsson et al., 1986, all but three implants showed an acceptable bone loss (annual bone loss of 0.2 mm except in the first year) [36]. The observed ΔMBL in the present study is consistent with the ΔMBLs documented in the literature in the context of short- and mid-term follow-ups. A systematic review [31] investigated ΔMBLs around MDIs retaining mandibular IODs. In this review, six of seven studies showed acceptable ΔMBLs below 1.5 mm, noting that the follow-up period was less than 5 years in the selected studies. The study that reported ΔMBLs of more than 1.5 mm evaluated implants in the maxilla and this may—according to the authors—have influenced the peri-implant outcomes. In a previous systematic review [14], the mean marginal bone loss in mini implants with a diameter of less than 3 mm was reported to be between 0.6 mm and 1.43 mm. Although these data only relate to short- and medium-term outcomes, this range can also be seen in the present study. While the average bone loss was within acceptable limits in the first follow-up period, a mostly constant bone level was recorded at the 10-year examination. Out of the total of 56 implants examined, 7 implants had ΔMBLs higher than −2 mm. Within this group, only two implants showed a ΔMBL exceeding −3 mm.

The clinical relevance of the presence of keratinized mucosa (KM) for maintaining the stability of the peri-implant hard and soft tissue is controversially discussed in the literature [37,38]. No influence of the presence of KM could be demonstrated in either the 5-year or the present 10-year study results. As for the other clinical parameters, the present investigation showed an increased incidence of plaque and BOP values compared with the 5-year follow-up [22]. Considering the age of the patients at the 10-year follow-up both in the younger and older groups (median age: 74 and 82 years), these findings are in agreement with other studies suggesting that advanced age as well as wearing IODs is related to higher BOP and plaque levels [39,40,41]. Because of the deterioration of vision, dexterity, and oral tactile perception with increasing age, there is less awareness of the presence of plaque and food debris in the mouth [42], which thereto leads to higher plaque values. Based on the above-mentioned considerations, the clinical outcomes of the present study are clinically acceptable. 

The marginal bone loss in old compared to young cohorts is diversely discussed in the literature. A systematic review by Schimmel et al. [43] found no evidence of higher implant failure rates or increased bone loss around implants in elderly patients compared to younger cohorts. Clinical studies could even observe lower ΔMBLs in the elderly than in younger patients [44,45]. Although the mechanism and details are not yet fully understood [46], there is increasing evidence that the immune response changes with age and that alterations in neutrophils and macrophages as well as inflammatory phagocytes play an important role [47,48]. In addition to the findings on age-related changes in the immune system, termed immunosenescence, there are also suggestions that low masticatory forces could be responsible for lower ΔMBLs [22]. Contrary to these outcomes, a large epidemiological study [49] attributed marginal bone loss around dental implants to time in function rather than age. Both the results of the 5-year follow-up of the same study cohort [22] and the results in the present 10-year study demonstrated significantly lower ΔMBLs in older participants and are thus consistent with findings in the literature. 

The most frequently mentioned technical complications in implant-supported overdentures are matrix loosening and fractures of the overdenture, regardless of the implant diameter used [31,50]. If fractures occur, they are usually located near the matrices and can be accompanied by matrix loosening. This correlation was shown to be even significant by a recently published cohort study, comparing maintenance and risk factors for fractures of IODs using conventional diameter implants or MDIs. Their findings suggest that, regardless of the implant diameter, matrix loosening represents a risk factor for IOD fractures [50]. Up to the 5-year follow-up, in 35% of the overdentures, fractures were observed. All of them occurred near the matrix area. After reinforcement with a metal framework at the lingual aspect, no further fractures were found [22]. Within the limitations of the present study, no technical complications were detected at the 10-year follow-up appointment. An overdenture survival of 90.58% for overdentures retained by MDIs was reported in a systematic review of complete overdentures retained by MDIs by Lemos et al. [31]. This is in agreement with the present observation, where the overdentures showed a 93% survival rate. However, in the current study, no reinforcements were integrated into the IODs, which might contribute to the relatively high incidence of denture base fractures in the beginning. 

To the best of our knowledge, the present investigation is the first longitudinal observational study reporting 10-year results of one-piece MDIs. As described in the 5-year follow-up report [22], there is only one prospective study with a follow-up of ≥5 years reporting on peri-implant MBLs. However, in contrast to this study, the authors used two-piece implants with a diameter of >2 mm [19]. The main outcome of the present study was to assess the temporal changes in the ΔMBL around one-piece MDIs and determine the differences between the first and the last five years after placement. With the usage of customized radiological splints, standardized radiograph recordings for each individual could be taken and compared with the pre-existing data to enable an accurate evaluation of the MBL. Furthermore, the measurements were performed separately by two clinicians and twice at two different time points. Although the MBL measurements differ significantly between clinicians (*p* = 0.013), as shown by the interrater reliability analysis, the estimated mean difference of 0.03 mm can be considered clinically irrelevant. 

The limitations of the present study include its small number of participants, the involvement of a single center, the lack of a control group, missing information on the history of periodontal disease and smoking habits, and the missing sample size calculation. Over the course of 10 years, 6 patients dropped out, which led to 14 included participants in the present study. A dropout rate of 30% over a 10-year follow-up period can be considered a success, especially when working with older participants who may have a higher dropout rate due to general health problems. However, the small number of participants represents a clear limitation of this study, which is why the results should be interpreted with caution. Another limitation is the fact that most of the patients included in the present study had their regular follow-ups at their private practices. Consequently, it is not feasible to evaluate the frequency of maintenance, complications, and dental hygiene recalls. 

## 5. Conclusions

Within the limitations of this non-comparative longitudinal observational study (especially the lack of a control group and the small sample size), one-piece MDIs retaining a lower IOD with O-ring attachments can be considered as an alternative treatment modality, as they provide high survival rates and good long-term outcomes for elderly patients with horizontally atrophic (‘knife-edged’) edentulous mandibles and poor oral hygiene. Further long-term follow-up studies with a higher number of subjects and with comparison to a control group are required to confirm the efficacy of mini implants, which will be the subject of future studies.

## Figures and Tables

**Table 1:** Overview of mean marginal bone level alterations.

Implant Position	3 Months	6 Months	12 Months	3 Years	5 Years	10 Years
34	−0.58 ± 0.62	−0.68 ± 0.66	−0.64 ± 0.62	−1.00 ± 0.88	−1.26 ± 0.89	−1.42 ± 0.88
32	−0.61 ± 0.42	−0.79 ± 0.62	−1.03 ± 0.67	−1.19 ± 0.70	−1.43 ± 0.73	−1.06 ± 0.53
42	−0.70 ± 0.52	−0.69 ± 0.61	−0.71 ± 0.79	−0.96 ± 0.79	−1.13 ± 0.73	−1.04 ± 0.58
44	−0.54 ± 0.39	−0.49 ± 0.43	−0.56 ± 0.62	−0.68 ± 0.73	−0.89 ± 0.75	−0.95 ± 1.08
Overall	−0.61 ± 0.49	−0.66 ± 0.58	−0.74 ± 0.68	−0.96 ± 0.78	−1.18 ± 0.78	−1.12 ± 0.80

**Table 2:** Analysis of keratinized mucosa.

Presence of Keratinized Mucosa	Implants (*n* = 56)	ΔMBL ≤ 2 (%)	ΔMBL 2–4 mm (%)
yes	36	86.1	13.9
no	20	90.0	10.0

ΔMBL in relation to the presence of keratinized mucosa (KM) at the 10-year follow-up.

**Table 3:** Overview of peri-implant parameters (pooled) at each follow-up appointment. Means (mv) and standard deviations (SDs).

Follow-Up (months)	PD (mm)	mPI (mv)	BOP (%)
3	3.19 ± 0.87	0.39 ± 0.42	64.3
6	3.39 ± 0.88	0.21 ± 0.31	50.0
12	3.50 ± 0.54	0.44 ± 0.42	42.9
36	2.00 ± 0.46	0.36 ± 0.50	21.4
60	1.67 ± 0.38	0.55 ± 0.37	64.3
120	2.17 ± 0.51	0.75 ± 0.35	92.9

Abbreviations: PD, probing depths; mPI, modified Plaque Index; BOP, bleeding on probing. Note: Values for BOP > 0 (for at least one implant, all implants per participant pooled).

## Data Availability

The raw data supporting the conclusions of this article will be made available by the authors on request, including respective authorization by an ethics committee.
